# Supplementary material for: Cost-effectiveness analysis protocol of the Smart Triage program: A point-of-care digital triage platform for pediatric sepsis in Eastern Uganda
Source: PLoS One. 2021 Nov 17;16(11):e0260044. doi: 10.1371/journal.pone.0260044 (PMC8598020; doi:10.1371/journal.pone.0260044)
Supplement: S1 Table — (DOCX) [file pone.0260044.s001.docx]

Table 1 Key Assumptions of the Decision Analytic Model

| **Assumption** | **Rationale** |
| --- | --- |
| Smart Triage exerts its strongest effects on mortality and short-term morbidity within the initial 7 days. | Smart Triage facilitates an acute intervention to target short-term outcomes (sepsis-induced mortality and morbidity during the immediate illness) and will likely exert its strongest effects in this timeframe. The model may be updated when long-term disability data and disability weights for sepsis survivors become available in LMICs. |
| The odds ratio of death for receiving a sepsis bundle within 1 hour in high-income setting may be used to link the Smart Triage study’s primary outcome to mortality in an LMIC setting. | LMIC-specific data in this regard is not yet available. However, due to various factors predisposing LMIC patients to a higher disease severity upon presentation and the lack of expensive intensive care support, a timely sepsis bundle may be even more crucial and exert a stronger effect in these patients. Our use of a high-income setting odds ratio may therefore not only be appropriate but a conservative estimate of the true value for LMICs. Please see the “discussion” section for details. Our model may be updated with an LMIC-specific odds ratio when available. |
| Costs of care in pneumonia can be used to approximate costs of care in sepsis. | Detailed costing data on sepsis-specific healthcare utilization are not available in Uganda, to our knowledge. Pneumonia features similar healthcare needs as sepsis (antibiotic, oxygen, fluids) and may progress to sepsis. Therefore, we will use previously published Ugandan costing data for pneumonia to approximate the care costs of sepsis ([22](#_ENREF_22)), and update our model with all-cause sepsis costs when available. |
| The cost of death is taken to be zero in our model. (Note that care costs leading up to death are not taken to be zero and are counted in our costing data.) | In costing studies ([24](#_ENREF_24)), OOP costing for death, such as funeral expenses, was foregone due to emotional trauma experienced by caregivers when asked such questions. Therefore, this data is not available. |
| Estimates of life expectancy from the World Health Organization, used to calculate YLL averted, representatively captures individuals with a history of childhood infection or sepsis. | Sepsis survivors are likely well-represented in census-based estimations of population life expectancy given a high childhood incidence of sepsis in Uganda. In addition, patients presenting to JRRH with infection vary widely in their initial severity of disease, and given JRRH’s wide catchment area, likely reflects the spectrum of childhood infection severity. Therefore, use of census-based estimates of life expectancy to calculate YLL averted for the JRRH sample seems appropriate. |
