# Supplementary material for: Cost-effectiveness analysis protocol of the Smart Triage program: A point-of-care digital triage platform for pediatric sepsis in Eastern Uganda
Source: PLoS One. 2021 Nov 17;16(11):e0260044. doi: 10.1371/journal.pone.0260044 (PMC8598020; doi:10.1371/journal.pone.0260044)
Supplement: Attachment — Submitted filename: PONE-D-21-21174_reviewer-commentsMA.docx [file pone.0260044.s002.docx]

PONE-D-21-21174_review comments

Manuscript title: Cost-effectiveness analysis protocol of the Smart Triage program: a point-of-care digital triage platform for pediatric sepsis in Eastern Uganda

The manuscript reports a study protocol. Sepsis is an important cause of mortality in children and early treatment results in better outcomes. ETAT and other guidelines when properly implemented show good predictive power for mortality and improved clinical outcomes.

The SMART Triage program tackles guideline complexity to predict, at admission, those children most likely to deteriorate. The SMART triage program consists of a mobile app linked to a dashboard and a locally hosted Bluetooth treatment tracking mechanism to improve patient organization.

The study is part of a larger study examining pediatric sepsis treatment and is done in Jinga Teaching and Referral Hospital in Uganda. Figure 1 is a useful aid in understanding where the data analysis decision points will be made.

The decision analytic model which includes a societal perspective is a valuable inclusion as out of pocket costs calculations recognize that patients’ bear a sizable portion of health care costs.

Previously published secondary data will be used to link healthcare utilization with costs and intermediate outcomes with mortality. This data is in the Harvard Dataverse repository. It requires permissions for downloading and as such, it was not available to the reviewer in a timely fashion. That said, the variables for calculation of costs seems reasonable based on the brief description available.

Monte Carlo simulations seem very appropriate for this type of analysis-though this type of analysis is outside my specific areas of expertise.

Comments:

This protocol is clearly written. Strengths and limitations are well stated. A major strength is the linking of the sepsis bundle within one hour to longitudinal clinical data and including the 7-day post discharge outpatient time frame. Another strength is the inclusion of cost data from out of pocket expenditures.

1. Please consider a wording change-(patient **care** organization may be better wording than patient organization)
2. The asserted high follow up rate in the pilot data is a very reassuring, but also quite uncommon (98.5%). Please describe what methods are used to ensure so little loss to follow up.
3. YLL averted is measured by a comparison of data from Jinga hospital where SMART Triage program in utilized (intervention) + one Kenyan hospital and data from one hospitals in Kenya (control).

Have the authors considered using a within Jinga hospital pre intervention time frame as a control for that comparison as well? YLL averted is the critical variable on which all other cost data is framed. Variation in implementation of the ETAT protocol between countries may be greater than hoped and could influence assessment of YLL averted.

Recommendation: Accept with minor revision
