# Supplementary material for: Cost-effectiveness analysis protocol of the Smart Triage program: A point-of-care digital triage platform for pediatric sepsis in Eastern Uganda
Source: PLoS One. 2021 Nov 17;16(11):e0260044. doi: 10.1371/journal.pone.0260044 (PMC8598020; doi:10.1371/journal.pone.0260044)
Supplement: Attachment — Submitted filename: R1_Response letter 12Oct2021.docx [file pone.0260044.s003.docx]

October 13, 2021

To

The Editor

PLOS ONE

Subject: PONE-D-21-21174: Cost-effectiveness analysis protocol of the Smart Triage program: a point-of-care digital triage platform for pediatric sepsis in Eastern Uganda.

Dear Editor,

I am writing in reference to the reviewers’ comments received on September 2^nd^ 2021, for the aforementioned manuscript under your consideration for publication in PLOS ONE. We found these comments very helpful in improving the overall quality of our manuscript.

For your convenience, all changes to the manuscript have been cited in this response letter, with additions highlighted in yellow. Also, the amendments are highlighted as track-changes on the revised manuscript herewith attached (R1_manuscript_tracked_changes_12Oct2021) for your perusal.

Thank you for your consideration,

Best regards.

**In response to the academic editor:**

1. *Please ensure that your manuscript meets PLOS ONE's style requirements, including those for file naming.*

Authors’ response: We have ensured that the manuscript meets PLOS ONE's style requirements.

2. *We note that the grant information you provided in the ‘Funding Information’ and ‘Financial Disclosure’ sections do not match.*

*When you resubmit, please ensure that you provide the correct grant numbers for the awards you received for your study in the ‘Funding Information’ section.*

Authors’ response: We have ensured that the information in both the ‘Funding Information’ and ‘Financial Disclosure’ section are matching and correct.

3. *Please review your reference list to ensure that it is complete and correct. If you have cited papers that have been retracted, please include the rationale for doing so in the manuscript text, or remove these references and replace them with relevant current references. Any changes to the reference list should be mentioned in the rebuttal letter that accompanies your revised manuscript. If you need to cite a retracted article, indicate the article’s retracted status in the References list and also include a citation and full reference for the retraction notice.*

Authors’ response: We have ensured that our reference list is correct.

**Responses to reviewer #1:**

*Reviewer #1: Thanks for the opportunity to review this manuscript entitled "Cost-effectiveness analysis protocol of the Smart Triage program: a point-of-care digital triage platform for pediatric sepsis in Eastern Uganda". This is a protocol for an important research question. Overall, the manuscript is well written with enough details in different sections. Tables are informative. Below are comments/concerns for the authors to consider.*

1. *Include a results section with expected outcomes and results to be reported*

Authors’ response: We have added the following “Results to be Reported” section in the revised manuscript:

The proportion of children who receive a sepsis bundle of care within one hour will be reported for both the pre-and-post intervention phases. Likewise, all other probabilities and cost inputs used in the model will be reported. The results of the Monte Carlo simulation will be presented on a cost-effectiveness plane to illustrate the percentage of incremental cost-effectiveness ratios (ICERs) in any of the four quadrants (i.e., ICERs falling in the North-east quadrant indicate higher costs and higher benefits, or ICERs in the North-west quadrant indicate higher costs and lower/poor outcomes, or ICERs in the South-east quadrant indicate lower costs and higher benefits, or ICERs in the South-west quadrant indicate lower cost and lower/poor outcomes). We will summarize these results on a cost-effectiveness acceptability curve, which will illustrate the confidence of Smart Triage being cost-effective at different thresholds of willingness to pay per YLL-averted. All results will be reported in multiples of Uganda’s GDP and United States dollars (USD) for ease of interpretability by local policymakers and the international research community.

Authors’ response: Of note, the following sentences were deleted from their respective original sections and incorporated into the “results to be reported” section:

From “Materials and Methods” – “Model Inputs” – “Adjusting Costs for Inflation”: We will report our results both in terms of Uganda’s GDP and in United States dollars (USD) for ease of interpretability by local policymakers and the international research community.

From “Materials and Methods” – “Model and Parameter Uncertainty” - “Probabilistic Sensitivity Analysis”: The results of the Monte Carlo simulation will be represented on a cost-effectiveness plane, and the confidence of cost-effectiveness at various thresholds of willingness to pay will be illustrated on a cost-effectiveness acceptability curve.

2. *Include a conclusions section*

Authors’ response: We have added the following “Conclusions” section in the revised manuscript:

This study concludes the importance of conducting a cost-effectiveness analysis of health technologies/interventions such as Smart Triage for the population at scale, especially in resource-constrained settings. To complement the Smart Triage interventional trial, aimed at providing expedited sepsis treatment in children, our study offers a methodological framework for cost-effectiveness analysis of the platform. Our study findings, together with subsequent analyses modelling budget impact and scale-up, will underscore financial implications for future modifications to the Smart Triage platform. In addition to the primary clinical evidence, the economic impact will inform policy decisions for scale-up to the district and national levels. This protocol may also serve as a template for the economic evaluation of similar technologies in LMICs.

3. *Discuss alternative strategies to address some potential limitations*

Authors’ response: Regarding the use of high-income country linkage data to link timely sepsis bundle with mortality in an LMIC setting, we have made the following addition (Discussion – limitations of study – first paragraph):

“Therefore, as a corollary, not only is our substitution with high-income setting linkage data appropriate, but a future repeated analysis with LMIC-specific data may reveal even greater cost-effectiveness than our proposed analysis. On the other hand, if we had foregone linking timely sepsis bundle to mortality due to the current unavailability of LMIC-specific linkage data, our analysis would generate only the cost per child receiving timely bundle as opposed to cost per YLL-averted, with a significantly reduced clinical interpretability.”

Regarding our foregoing of calculating disability adjusted life years due to the lack of LMIC-specific disability weights, and using years of life lost averted instead, we have made the following addition (Discussion – limitations of study – second paragraph):

“Another area where LMIC-specific evidence is lacking is long-term quality-of-life data and corresponding disability weights in sepsis survivors. Therefore, we are unable to estimate years lived with disability for calculating DALYs. Extrapolating quality-of-life evidence from high-income settings into the LMIC setting would be inappropriate given vast differences in social support systems. We also lacked the resources to carry out our own LMIC-based valuation study where different health states are methodically assigned disability weights. When such evidence with appropriate valuation data becomes available in the LMIC setting, long-term outcomes may be incorporated into our economic evaluation through a Markov model.”

4. *Discussion, the authors should expand and elaborate more on how their findings support or contrast available literature and provide suggestions for future research directions that would address existing knowledge gaps.*

Authors’ response: Thank you for this suggestion. While we do not yet have results with which to compare to existing literature, we have made the following changes to highlight our analysis in the context of existing literature in high-income settings (Discussion – Strengths & Implications of Study – second paragraph):

To our knowledge, our analysis is the first proposed study to employ a decision-analytic model in evaluating the cost-effectiveness of a sepsis program focused on triage in an LMIC setting. This technique allows for a more comprehensive evaluation of cost-effectiveness compared to previous economic analyses of LMIC triage programs (1, 2), and allows for comparison of cost-effectiveness to analyses of sepsis triage programs in high income settings (3, 4).

Regarding future directions, we have made the following changes to highlight the importance of a budget impact analysis to fill the knowledge gap left by a cost-effectiveness analysis (Discussion – Limitations – last paragraph):

Lastly, while our cost-effectiveness analysis will be an important first step in shaping local policy towards scaling up of Smart Triage should cost effectiveness be demonstrated, our analysis does not take into account the impact of scale up on issues such as reallocation of resources, impact on healthcare budget given sepsis’ burden of disease (5), and multicenter scale-up costs (6). To this end, future directions of study may include a budget impact analysis factoring in scale-up costs, the number of centers for scale up, and the large burden of disease of sepsis in these centers.

**Responses to reviewer #2**

*Reviewer #2: Manuscript title: Cost-effectiveness analysis protocol of the Smart Triage program: a point-of-care digital triage platform for pediatric sepsis in Eastern Uganda*

*The manuscript reports a study protocol. Sepsis is an important cause of mortality in children and early treatment results in better outcomes. ETAT and other guidelines when properly implemented show good predictive power for mortality and improved clinical outcomes.*

*The SMART Triage program tackles guideline complexity to predict, at admission, those children most likely to deteriorate. The SMART triage program consists of a mobile app linked to a dashboard and a locally hosted Bluetooth treatment tracking mechanism to improve patient organization.*

*The study is part of a larger study examining pediatric sepsis treatment and is done in Jinga Teaching and Referral Hospital in Uganda. Figure 1 is a useful aid in understanding where the data analysis decision points will be made.*

*The decision analytic model which includes a societal perspective is a valuable inclusion as out of pocket costs calculations recognize that patients’ bear a sizable portion of health care costs.*

*Previously published secondary data will be used to link healthcare utilization with costs and intermediate outcomes with mortality. This data is in the Harvard Dataverse repository. It requires permissions for downloading and as such, it was not available to the reviewer in a timely fashion. That said, the variables for calculation of costs seems reasonable based on the brief description available.*

*Monte Carlo simulations seem very appropriate for this type of analysis-though this type of analysis is outside my specific areas of expertise.*

*Comments:*

*This protocol is clearly written. Strengths and limitations are well stated. A major strength is the linking of the sepsis bundle within one hour to longitudinal clinical data and including the 7-day post discharge outpatient time frame. Another strength is the inclusion of cost data from out of pocket expenditures.*

1. *Please consider a wording change-(patient care organization may be better wording than patient organization)*

Authors’ response: Thank you for this suggestion. We have made the wording change in the manuscript.

2. *The asserted high follow up rate in the pilot data is a very reassuring, but also quite uncommon (98.5%). Please describe what methods are used to ensure so little loss to follow up.*

Authors’ response: We have made the following additions to describe methods to minimize loss to follow-up (Discussion – Strengths & Implications of Study – First paragraph):

Finally, our unpublished study data thus far indicates a high follow-up rate of 98.3%, minimizing the impact of incomplete outcome data in our analysis. As we relied exclusively on telephone follow-up, we optimized follow-up through rigorous checks for phone number accuracy, repeat calls to unreached participants through a standardized process, and use of shared phone numbers, such as that of a neighbor, should a personal number not be available, Our final follow-up rate will be published upon completion of the trial.

3. *YLL averted is measured by a comparison of data from Jinga hospital where SMART Triage program in utilized (intervention) + one Kenyan hospital and data from one hospitals in Kenya (control). Have the authors considered using a within Jinga hospital pre intervention time frame as a control for that comparison as well? YLL averted is the critical variable on which all other cost data is framed. Variation in implementation of the ETAT protocol between countries may be greater than hoped and could influence assessment of YLL averted.*

Authors’ response: Thank you for this inquiry. Our analysis is only confined to Jinja hospital, using the pre-implementation data at Jinja hospital as a control. However, this design was evidently not clearly conveyed by the original submission. We have therefore made the following addition for added clarity (Methods – Description of Parent Clinical Trial – first paragraph):

The Smart Triage trial was designed as a difference in differences trial, with a Kenyan site for control. However, we will restrict this cost-effectiveness analysis to the Ugandan site at JRRH, to minimize the impact on our analysis due to variations in baseline ETAT implementation between countries. Therefore, we will compare the post-intervention data at JRRH with that of the pre-intervention phase, with the latter serving as control for our analysis.

We have also made the following change under “Materials and Methods” – “Study Objectives” to reflect this design:

Calculate the incremental cost relative to years-of-life lost (YLL) averted from the Smart Triage program compared to the standard of care, using the pre-intervention phase at JRRH as the control.

**Response to reviewer #3**

*Reviewer #3: Thanks for the opportunity to review this interesting protocol. The protocol is well written and detailed, and I make a few suggestions that may make the work easier to follow.*

*Materials and Methods*

1. *I suggest you summarize the predictive model the Smart Triage is based on – unless its proprietary. It would be helpful for readers. If proprietary, please say so.*

Authors’ response: Thank you for this comment. The publication that gives specifics on Smart Triage’s predictive model is currently under review. We have made the following changes to give a summary of the model’s makeup (Introduction – Sepsis, The Importance of Triage, and The Smart Triage Program - third paragraph):

The Smart Triage program (7) tackles guideline complexity via a prediction model for admission to facilitate the identification of children at risk of deterioration. This model classifies children into three levels of risk categories, using clinical variables found in ETAT and other triage studies that are then used as inputs in an algorithm derived from machine learning techniques. Our methods for initial consideration of variables have been published (8), and the details of the final algorithm is under peer review. This model was developed using data from the trial’s pre-intervention phase, and has been integrated into a mobile application to reduce cognitive error and training time compared to a manual triage process.

2. *Some abbreviations are still not written in full at first use e.g. OOP*

Authors’ response: Thank you for noting this issue. We have made the necessary corrections.

3. *Description of parent trial – provide more detail on the trial. Are patients randomized? How? Are all patients at the outpatient department screened and enrolled or is there a sampling framework? Does the trial collect data on hard outcomes such as mortality, even if it is not powered to assess them?*

Authors’ response: We have made the following highlighted additions to the “Description of parent clinical trial” section to address these inquiries:

The Smart Triage program and a corresponding clinical trial will be implemented in the pediatric outpatient department at JRRH in Uganda, which serves children up to age 18 presenting from the community at various degrees of disease severity. The outpatient department serves as a gateway to admission if necessary. All presenting children, except those with scheduled appointments, are eligible to participate. However, enrollment of all children is not possible due to a high patient load, and therefore a sampling framework as published in the parent trial’s protocol is used to obtain a representative sample (7). The Smart Triage trial was designed as a difference in differences trial, with a Kenyan site for control. However, we will restrict this cost-effectiveness analysis to the Ugandan site at JRRH, to minimize the impact on our analysis due to variations in baseline ETAT implementation between countries. Therefore, we will compare the post-intervention data at JRRH with that of the pre-intervention phase, with the latter serving as control for our analysis.

The primary outcome of the Smart Triage trial is the difference in the proportion of children who receive a sepsis bundle of care within one hour before and after Smart Triage’s implementation. Enrolled children will be followed up to assess in-hospital mortality (if admitted), 7-day post-discharge mortality, and 7-day post-discharge healthcare utilization rates.

4. *It seems that the methods make a fundamental assumption – that more children receiving the sepsis bundle is good, even if it is not indicated. Please clarify.*

Authors’ response: We have made the following addition under “Description of parent clinical trial” to clarify that we do not make this assumption:

Of note, Smart Triage does not aim to indiscriminately increase the number or proportion of children receiving sepsis bundle. Indication for sepsis treatment is determined by local clinicians both before and after Smart Triage implementation. Smart Triage simply facilitates the timelier delivery of sepsis treatment, if deemed indicated by local clinicians, through a data-driven quality improvement process.

5. *Pneumonia patients often require imaging. Will the cost data be item by item, allowing the exclusion of items that may not be relevant? Or are we assuming that it the costs where pneumonia and sepsis differ may not meaningfully matter since pneumonia often leads to sepsis too.*

Authors’ response: We have made the following addition under “Discussion” – “Strengths & Implications of Study” to clarify this inquiry:

While healthcare utilization and associated costs of pneumonia may not be identical to sepsis, the two disease processes have significant overlap as pneumonia may progress to sepsis if sub-optimally treated, particularly in resource-constrained settings. Therefore, we chose to use secondary data (proxy variables) for pneumonia care costs in their entirety, rather than an item-by-item selection of costs.

This addition elaborates on and replaces this sentence from the same location in the original submission:

While care costs of pneumonia may be slightly lower than that of sepsis due to the former involving only a single organ system, many cases of pneumonia may nonetheless progress to sepsis and accrue the associated increase in care costs

6. *Are the cost data from public or private settings? How much variation is factored in?*

Authors’ response: We have made the following addition (Methods – Model inputs – Program costs) to clarify this question:

As Smart Triage is being implemented at JRRH, a public center, program costs borne by the research team will be regarded as costs from the government perspective. We will assume a +/- 25% variation in a deterministic distribution to account for uncertainty in a probabilistic sensitivity analysis.

7. *The models assume that the life expectancy of sepsis survivors is similar to the life expectancy of the general population. Without data to back it up, this assumption seems fraught.*

Authors’ response: Of note, we are not assuming that having sepsis would have no impact on one’s potential life expectancy. We are using WHO’s population life expectancy estimate in Uganda to calculate how many years of life would be lost if a patient died from infection, and therefore how many years of life lost (YLL) would be averted if this death did not occur. The individuals visiting Jinja hospital’s outpatient department have varying degrees of presenting severity (i.e. only a small proportion are initially septic, most are not at arrival but may deteriorate). Most cases are suspected infection. Having such a wide range of presenting severity due to a large catchment area means that presenting patients at JRRH is quite representative of the range of childhood infection severities in Uganda. Given the high prevalence of childhood infection in Uganda, which includes sepsis, we believe that individuals with a history of such childhood infection would be representatively captured in the life expectancy estimate of WHO. It is with this rationale that we use the WHO estimate for life expectancy to calculate YLL. However, we realize that the wording in our original submission is misleading. To avoid misleading readers into thinking that we assume the life expectancy of sepsis survivors and the general population are the same, we have modified the relevant row in table 1 as follows:

Original submission:

| Overall population life expectancy, used in our model to calculate years of life lost from mortality, is representative of a sepsis survivor. | Life expectancy data specific to sepsis survivors are not available in Uganda to our knowledge. However, sepsis survivors are likely well-represented in census-based estimations of population life expectancy given a high childhood incidence of sepsis (26). Therefore, using the overall population life expectancy to calculate years of life lost should reasonably approximate that of sepsis survivors. |
| --- | --- |

Revised submission:

| Estimates of life expectancy from the World Health Organization, used to calculate YLL averted, representatively captures individuals with a history of childhood infection or sepsis. | Sepsis survivors are likely well-represented in census-based estimations of population life expectancy given a high childhood incidence of sepsis in Uganda. In addition, patients presenting to JRRH with infection vary widely in their initial severity of disease, and given JRRH’s wide catchment area, likely reflects the spectrum of childhood infection severity. Therefore, use of census-based estimates of life expectancy to calculate YLL averted for the JRRH sample seems appropriate. |
| --- | --- |

References

1.	Robison JA, Ahmad ZP, Nosek CA, Durand C, Namathanga A, Milazi R, et al. Decreased pediatric hospital mortality after an intervention to improve emergency care in Lilongwe, Malawi. Pediatrics. 2012;130(3):e676-e82.

2.	Barasa EW, Ayieko P, Cleary S, English M. A multifaceted intervention to improve the quality of care of children in district hospitals in Kenya: a cost-effectiveness analysis. PLoS medicine. 2012;9(6).

3.	Noritomi DT, Ranzani OT, Monteiro MB, Ferreira EM, Santos SR, Leibel F, et al. Implementation of a multifaceted sepsis education program in an emerging country setting: clinical outcomes and cost-effectiveness in a long-term follow-up study. Intensive care medicine. 2014;40(2):182-91.

4.	Afshar M, Arain E, Ye C, Gilbert E, Xie M, Lee J, et al. Patient outcomes and cost-effectiveness of a sepsis care quality improvement program in a health system. Critical care medicine. 2019;47(10):1371-9.

5.	Donaldson C, Currie G, Mitton C. Cost effectiveness analysis in health care: contraindications. Bmj. 2002;325(7369):891-4.

6.	Marseille E, Larson B, Kazi DS, Kahn JG, Rosen S. Thresholds for the cost–effectiveness of interventions: alternative approaches. Bulletin of the World Health Organization. 2014;93:118-24.

7.	Mawji A, Li E, Komugisha C, Akech S, Dunsmuir D, Wiens MO, et al. Smart triage: triage and management of sepsis in children using the point-of-care Pediatric Rapid Sepsis Trigger (PRST) tool. BMC Health Services Research. 2020;20(1):1-13.

8.	Mawji A, Li E, Chandna A, Kortz T, Akech S, Wiens MO, et al. Common data elements for predictors of pediatric sepsis: A framework to standardize data collection. PloS one. 2021;16(6):e0253051.
